# Assessment of ECG and respiration recordings from simulated emergency landings of ultra light aircraft

**DOI:** 10.1038/s41598-018-25528-z

**Published:** 2018-05-08

**Authors:** Ondřej Bruna, Tomáš Levora, Jan Holub

**Affiliations:** Department of Measurement, Faculty of Electrical Engineering, Czech Technical University in Prague, Technická 2, Prague, 166 27 Czech Republic

## Abstract

Pilots of ultra light aircraft have limited training resources, but with the use of low cost simulators it might be possible to train and test some parts of their training on the ground. The purpose of this paper is to examine possibility of stress inducement on a low cost flight simulator. Stress is assessed from electrocardiogram and respiration. Engine failure during flight served as a stress inducement stimuli. For one flight, pilots had access to an emergency navigation system. There were recorded some statistically significant changes in parameters regarding breathing frequency. Although no significant change was observed in ECG parameters, there appears to be an effect on respiration parameters. Physiological signals processed with analysis of variance suggest, that the moment of engine failure and approach for landing affected average breathing frequency. Presence of navigation interface does not appear to have a significant effect on pilots.

## Introduction

Manned systems are far more complex and far more advanced than they were two decades ago. Therefore a lot of attention has been paid to evaluation of situational awareness and workload imposed on operators, pilots, drivers and other professions while interacting with some kind of system^1–9^. A train is able to travel autonomously, but there is still a person required to ensure safety, especially in unforeseen situations. Unmanned/unattended vehicles are very advanced, but in crucial areas there are still humans who are in control. There is still a pilot needed in the transport airplane, even though some airports allow for fully automated landings and approaches. The pilot is there to ensure the right decision is made in unpredictable situations–for example bad weather conditions. A person is believed to be able to deal with the situation successfully. The situation is not different for small aircraft (light sport aircraft, ultra light aircraft), where the pilot receives less training and has less experience when flying for the first time. According to European Aviation Safety Agency (EASA), controlled flight into terrain is the fifth most common cause of accidents^10^.

It is known, that in some cases even human decision making fails. Most known causes are the lack of situational awareness, and lack of information due to fatigue or stress^11,12^. Fatigue or stress can affect the way people make decisions and react to inputs. As a result, there are systems which are meant to help maintain situational awareness and to provide well organized, easy to understand, and at the time being, the most necessary information^2,13–18^. Some of these systems aim specifically at helping pilots with emergency landings^15,17,19^.

In the scope of this article, the focus will be on small manned aircraft (mainly pilots of ultra-light aircrafts - ULA) in general aviation, which encounters problems with the engine, resulting in engine failure and a forced emergency landing. Pilots of the damaged aircraft can make a wrong decision on where to perform an emergency landing or may fail in making a correct estimation, eventually in performing the final approach. Pilots of ULA receive reduced training and have less flight hours and less opportunity to fly, and therefore lack experience in comparison to their professional counterparts.

Due to fast advancing technologies it is possible to see equipment such as glass cockpits in small aircrafts, which is very similar to that found in large civilian airliners. New technologies make it possible to implement new systems easily and in a very short time. Some pilots use their tablets and smart phones on board the airplane as a primary flight display or synthetic vision display. In this paper we present an emergency landing assistant and results from its testing with pilots. An experiment was conducted to measure physiological parameters to determine the differences between flight with an assistant and without. This can help us to understand how these systems help pilots to deal with emergency situations. The main questions we are asking are: What physiological parameters are sensitive to simulated emergency landing? and What is the effect of navigation instrument to these parameters?

## Design of Emergency Landing Navigation Assistant

### Description

Many small single engine aircraft pilots are not prepared and trained to cope with engine failure events; they are usually not trained on glider airplanes so they think in different ways in such situations as they lack experience typically gained from gliding. A distracted pilot can respond very rashly and can make maneuvers that lead to a crash landing. To help a pilot to deal with such situation, an emergency landing assistant is proposed. The assistant takes advantage of modern glass cockpit equipment (primary and secondary flight displays, synthetic vision system^14^) together with modern guidance systems^6,20,21^. The system activates in a state of emergency, and based on the remaining kinetic and potential energy it calculates a reachable area and searches for a safe landing site within this range.

The detection algorithm for this kind of emergency situation is simple and requires only an engine revolutions sensor. Revolutions below a certain threshold during flight (non-zero altitude above ground) will activate the assistant. When activated, an emergency landing runway would be searched for and chosen, and afterwards the flight path from actual aircraft position to desired runway would be planned and the navigation would start by displaying the selected navigation paradigm, which then would direct the pilot to the landing site. The selected configuration is intended to not only lead the pilot to the destination, but also prevent him from any maneuver on the edge of safety and stability.

### Landing Site Search and Selection Module

A more sophisticated method relies on image and terrain segmentation and processing. A camera can be used to provide data from the ground under the aircraft and based on image processing it can deliver target locations suitable for landing^17^. Some of the ground cover is more suitable for landing than others and it can be important information for a landing site voter. The intelligent camera module should not be an autonomous module and will use information from raster map processing. Map layers containing cities, bodies of water, forests, and agricultural land are very useful. They can help to determine exact locations of the fields. This method, combined with map layers processing, is believed to provide enough inputs for selection of a safe landing site^6^. The height map layer can help an algorithm to exclude areas that are too high.

### Path Planning Module

Flight path planning algorithms in avionics are used mainly in unmanned aircrafts. Modern algorithms are usually based on genetic principles^22,23^. Some approaches that were developed tested trajectory design for emergency landings specifically in^20,24^.

Published algorithms have usually one property in common–they use spline as curves for flight paths representation. Another approach uses way-points on which the pilot can be directed by the system. However navigation along lines and circles is implemented in the assistant presented in this paper. The major concern regarding splines is that they would require constant attention. In terms of way-points, the concern is that the energy might be wasted by inadequate maneuvers when turning the airplane. Circles and lines appear to be a good compromise between these two options.

A very simple path planning algorithm has been implemented in the current version of the electronic emergency assistant. It plans a trajectory to the closest possible airport in reach and calculates the trajectory in a way that the pilot is lead to the runway threshold, taking the final approach into account. When the aircraft gets into an emergency situation, the maximal length of the trajectory to the ground is limited by the altitude, the airspeed and angle of descent. Using the speed polar characteristic and maximal lift-to-drag ratio the recommended airspeed can be determined^6^. According to current airspeed and altitude, minimal and maximal airspeed and corresponding to lift-to-drag ratio could be approximately determined. From this interval one point is selected and the desired flight path length should be moved closer to the path length at this point. Another variable should respect the angular turning length–a bigger radius and smaller heading difference are preferred due to safety reasons.

### Navigation

Flight path is a sequence of arcs and line segments, thus the navigation problem could be divided into two parts–navigation along lines and navigation along circles. In the implementation, line segments are defined by their end points and arcs are redundantly defined by their center, start point, end point and direction.

Several different navigation algorithms were implemented. The assistant allows guidance using several different configurations. One of them is “tunnel” or “highway in the sky” or “pavers”. This algorithm shows static shapes depending on the style; for tunnel it is rings along a desired flight path through which an aircraft should fly; for pavers the shapes, called pavers, are placed on the desired flight path. Both the tunnel and the pavers are shown at a short distance in front of the airplane and they move themselves along the flight path. This kind of navigation is quite easy to implement and it is a convenient method for guidance^25^.

Another navigation algorithm is based on commands provided to the pilot. The navigation module prepares advice to the pilot on where to fly–left, right, up, down, and action magnitude. The implemented algorithm calculates a so called meeting point that is defined as the intersection of the desired flight path and a circle with the radius of a distance in which the airplane exceeds the certain period. The intersection point in the direction of flight is chosen for navigation. The function of navigational command calculation should determine the distance of the aircraft position from the meeting point in both vertical and horizontal directions. Both distances are then displayed on the screen like vector commands. The vector is placed into the screen center and only the end point is plotted. In the vector end point, two crossing line segments are displayed. The cross consists of a horizontal (parallel to artificial horizon) line and a line perpendicular to the horizontal one.

The navigation procedure is a state machine running along the whole flight path. The state of the machine contains current flight path segment–line segment or arc. When the meet point reaches the end of the current shape, the state of the state machine switches to another one. While there is no meet point–ideally only when the aircraft is too distant from the flight path–the whole trajectory is recalculated.

## Methods

This investigation was performed at the Department of Measurement, Faculty of Electrical Engineering, Czech Technical University in Prague, Czech Republic. Ethical and regulatory classification was performed by the institutional review board and was approved by the faculty dean by a decision letter issued on 25/02/2014. The study met the ethical principles of the Declaration of Helsinki. All experiments were performed in accordance with the relevant guidelines and regulations. All involved subjects provided their written informed consent prior the experiment. There are no subject identifying details (HIPAA) in our contribution.

### Experiment design

The goal, as mentioned in the introduction, lies in performing experiments that will provide insight onto whether and how less skilled pilots are affected by a glass cockpit navigation instruments in the case of an emergency landing. Informed consent was obtained from all individual participants included in the study.

The tests used objective measures as well as subjective ones. The objective measures served to analyze the pilots’ performance in terms of flight safety and quality. It is defined by the basic flight rules taught in primary flight courses: maintain proper multiples of g, do not cross the air speed over the stated limit, do not go over the recommended pitch and roll rates and angles. Two rounds of tests were designed: firstly to evaluate the screen interface, and secondly to evaluate the performance of the pilots and their impression of the flight. Objective data allow for the assessment of each pilot in terms of abilities, skills and to score each pilot on his or her abilities.

The experiment consisted of two parts: (a) The first, presented pilots with different navigation configuration and let pilots perform a test flight, where they had a chance to get used to the environment and behavior of the simulator. After a test flight, pilots performed two flights, each with a different navigation paradigm and with the engine still running. These flights were important to prepare pilots for the following part and to understand their flight performance during normal conditions. (b) The second, pilots were given information that they will perform three flights, where they are supposed to start from one airport and land at another one. They also got a map to prepare for their trip. When the data was loading the pilots were asked which navigation paradigm they would prefer if they should encounter emergency conditions. Then the pilots took off, and based on random distribution, engine failure was generated in two out of three flights. During one flight the pilot did not have the emergency landing assistant available and had to rely on his own skills. The phases are divided into phase 1 - before failure, phase 2 - engine failure, phase 3 - soaring to the airport, phase 4 - approach and landing.

After the last flight pilots were presented with a simple questionnaire where they rated each segment of flight according to perceived level of stress. Ratings of the subjective questionnaires related to flight segment were from one to four with the following meaning: 1–Most stressed; 4–least stressed. Pilots were asked to rate on a Likert scale from 1 (not stressed at all) to 20 (extremely stressed) how stressful each flight was. In addition they rated on the same scale, how stressed they think they would be in a real situation.

### Apparatus

Subjective and physiological measures were used to assess stress and workload. The most often and discussed in previous research are electrocardiogram (ECG), electroencephalogram (EEG), electrooculogram (EOG), electromyogram (EMG), electrodermal activity (EDA), blood pressure (BP), photoplethysmography (PPG), pupil diameter (PD), heart rate (HR), heart rate variability (HRV), skin temperature, and respiration belt^26–32^.

For the purposes of this paper stress will be assessed based on ECG, EDA and respiration. Other means were not available at the moment of measurement. To record the data it was necessary to connect pilots with a logging device. The EEG was not measured, because the environmental conditions were too harsh and would not allow for safe data collection. Data were collected with BIOPAC, sampling frequency was 500 Hz.

In former experiments, verified methods to induce stress were used. Among the most common methods were public speaking, public arithmetic task, stroop color word test, cold pressor test, computer work and games^33^.

This paper relies on inducing stress by virtual reality flight in which the plane is damaged and the engine fails. The pilot then must perform an emergency landing. This event happens without the pilot being told beforehand.

The department is equipped with a 6DOF enclosed hydraulic simulator. Simulator was used for all experiments–it represented a virtual environment in which the pilots flew and fulfilled their tasks. The pilot had one display available with a Fresnel lens before him, where he could observe the environment. The other display, situated underneath the main, served as a electronic flight information system (EFIS) providing the pilot with all necessary information. Controls involved in the experiment were joystick, throttle, rudder pedals, and trim.

The software used for simulation was FlightGear 2.4. It is an open source program that is easy to modify and work with. The navigation assistant developed at the department was implemented as a separate program and run on another machine than the computer with simulation software. The landing assistant was used for all simulations to provide either emergency guidance or flight variables to the pilot. The goals were firstly to evaluate how a pilot interacts with the proposed navigation, and secondly to be able to evaluate their interaction and stress based on a simulated flight. From previous tests it was decided that the navigation will be provided by tunnel and cross on the EFIS screen.

### Scenarios

Only one person was tested at a time. Initially, the test subjects were given 10 minutes to practice with the simulator. During this time they were given simple tasks such as to descend to a certain flight level or to change heading and maintain it. The subjects were not required to takeoff with the aircraft, because the program started with the plane already flying at 4000 *ft* altitude. Once the subjects reported they were ready, the experiment proceeded to the next phase.

After warm up, pilots were given a task to follow the predetermined path that was presented to them on an EFIS screen in the form of a navigation metaphor: The test introduced two–cross and tunnel. Both flights were run with the engine on. The aircraft started flying at the same location and at the same altitude every time, and 15 seconds after the start, the navigation instrument activated. The pilot was supposed to follow the predetermined path from the starting point of the flight to the landing site as closely as he could. The trajectory was eight like loop with descending tendency. Each flight took approximately 10 minutes. After landing subjects reported first impressions and filled in prepared questionnaires. When finished, the next test followed. Once the last flight was done, the pilots were asked to fill in the overall assessment of flight interfaces and were interviewed. Pilots were also asked which metaphor they would use (if any at all) in case of navigation for emergency purposes. This navigation was then set-up for them.

Pilots were presented with an ICAO map of the Czech Republic and were given a task to prepare a navigation flight. They were instructed that this flight would be used for comparison with the two flights they had already flown. There were three flights ready and in two of them the engine would fail and the pilot would have to perform an emergency landing. In the case of an engine failure in one case the pilot had the navigation assistant available and in other he had to land on his own.

### Sample

The database was created using Google tools. Pilots registered into the database by filling in a simple screening questionnaire. The database allows us to filter the pilots according to several parameters. Our main aim was to recruit pilots who were not experienced and who were mainly flying aircraft for leisure. There was a special focus on pilots who had licenses for ultra-light aircraft, but other pilot licenses were also accepted.

The aim was to select younger pilots with less experience to whom the navigation assistant may be more useful.

Based on the database and people willing to get involved, this is the tested sample: 20 pilots aged from 21 to 48, with average of 30 years, were tested. The average number of flight hours was 200. The maximum amount of flight hours was 800. Some subjects had flown less than 100 flight hours. Some subjects reported that they had previous experience with emergency landing. Most of the pilots had a professional pilot license, but there were also some who had a glider license as well. These pilots were extremely useful when stating the drawbacks of our emergency landing assistant. In the selected pilots, some claimed to have previous experience with Garmin G-1000 or with other EFIS. Some pilots reported that they used the application in their tablets to serve the purpose of an EFIS. Some applications are known to be able to emulate an artificial horizon. Some pilots use even smart phones in order to emulate an EFIS.

### Independent Variable

There were two independent variables - availability of emergency landing assistant (availability had two levels - either the navigator was present, or not), and the second independent variable might be considered an engine failure. But for purposes of this paper only situations with engine failure are taken into account. Yet engine failure allows to create four segments in the flight, that are also considered independent variables. For each subject at least three flights were obtained, however; for evaluation, only two were taken, giving altogether 40 flights to evaluate.

### Dependent Measures

To determine stress levels different parameters were used. Signals from ECG, EDA and respiration were selected. For analysis several parameters from, time and frequency domain were chosen. Respiration based spectral power parameters from 0 to 2 Hz (divided into four intervals by 0.5 Hz) are annotated RF1, RF2, RF3, and RF4. Respiratory time domain parameters involved in this study were based on inter-breath intervals. Quantities used were average amplitude of minimum and maximum pairs in one breath cycle (FPVS), standard deviation of amplitude of single breath cycle (SDPVS), Average of breathing frequency (ABF), and Standard deviation of breathing frequency (SDBF).

To evaluate heartbeat, heart rate variability (HRV) was calculated based on RR intervals (temporal distance of two consecutive R peak intervals from QRS complex). Additional parameters involved standard deviation of RR intervals (SDNN), standard deviation of average RR intervals in 10-sec intervals in a segment (SDANN), squared root of average of squared difference of two consecutive RR intervals (RMSSD), and standard deviation of RR increments (SDSD). Frequency based measure used was a ration between high and low frequency band of HRV (LFHF).

Entropy measures^34^ were applied to evaluate additional information from signal. To evaluate entropy of HRV and respiration approximate entropy, sample entropy, and conditional entropy was used.

Electro-dermal activity (EDA) was collected to distinguish the contributions of sympathetic and parasympathetic systems. Heart variability and respiration parameters are measures of both systems. It is intended to evaluate EDA with additional parameters^35–38^ and compare the results. This will be subject of another study. All signals were extracted from the log file provided from BIOPAC Data acquisition and analysis system and evaluated by MATLAB.

From above mentioned measurements we expected to determine stress levels, but first all signals were evaluated separately to determine the proper parameters for evaluation. Stress is considered to be present when the frequency is getting higher and when the power is rising.

Measured values were crosschecked with the subjective rating of perceived stress in each phase of flight. Pilots ordered all four segments of flight from most stressful to the least stressful.

### Expected results

Two sets of data composed of flights with navigation after engine failure, and flights without navigation after engine failure are obtained from experiment. Each record in a set is divided into four phases based on events occurring. Sets are compared against each other and processed with analysis of variance applied to before mentioned parameters.

It is expected to observe increase of EDA, heart rate, respiration rate during the engine failure phase and possibly during approach and landing phase in all recorded sets. Comparison of the set with navigation against the set without navigation should demonstrate if there is any significant difference in parameters. It was expected that pilots would be likely to choose HITS (highway in the sky/tunnel) than cross for navigation purposes.

Subjective rating of stress is expected to mark the engine failure phase as the most stressful and the fist phase of flight (cruise) as least.

### Data availability

The datasets generated and analyzed during the current experiment are available from the corresponding author on reasonable request.

## Results

### Subjective Data

The subjects rated how stressed they felt in case of an emergency landing without emergency navigation and in case of landing with it. The flight was separated into four phases and pilots were asked to order phases from the most stressful to the least for both flights. This rating was then analyzed against physiological data.

**Figure 1:**
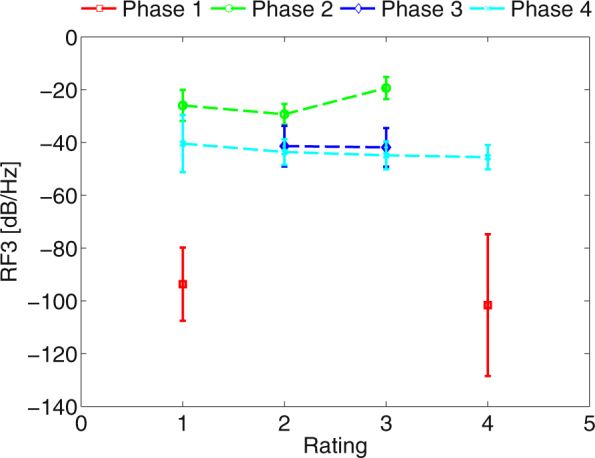
Standard deviation and mean of RF3 against pilots perceived stress rating (1–most perceived stress, 4–least perceived stress) across all flight phases (1–before engine failure, 2–engine failure, 3–glide, 4–approach and landing).

In Fig. 1 can be seen, that none of the pilots rated phase before failure (phase 1) as 2nd or 3rd most stressful. On the other hand the gliding phase (phase 2) was rated only with score 2 and 3. The moment of engine failure was never rated by pilots as the least stressful. Phase before engine failure was rated with scores 1 and 4. The subjects were divided between those who considered the first phase as most stressful or as the least stressful. It could be also due to the nature of the experiment. Subjects might suspect it is expected from them to chose the landing part as the most stressful. Physiological data show RF3 parameter of the first phase to be 30 dB/Hz lower than other phases. This is also confirmed by statistical data analysis.

Multiple analysis of variance with 2 × 4 design for the two groups of flights (flight with navigation and without it) and for four phases of flight (before engine failure, during engine failure, glide without engine, approach and landing) is used to determine which variables have significant effect on the mean. To determine statistical significance of differences between individual conditions such as rating, phase or flight, multiple comparison tests were used based on Student’s t-test with Bonferroni correction.

**Figure 2:**
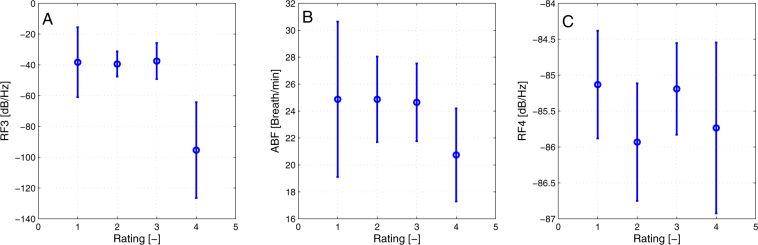
Mean and standard deviation of RF3, ABF, and RF4 parameters with respect to rating (1–most perceived stress, 4–least perceived stress).

Statistically significant results were observable for parameters RF4, RF3, and ABF. Results of MANOVA show that only rating had significant effect on the parameter’s mean. The flight type does not appear to have and effect. Rating affects the RF3 mean with p = 1.033e-13, ABF with p = 0.005, and RF4 with p = 0.0154. The multiple comparison test show that the mean of rating 4 (least stressful) of RF3 parameter is significantly different on level *α* = 0.05. On the same level of significance is statistically different mean of rating 4 of ABF parameters and mean of rating 2 of RF4 parameter. Although as can be seen from Fig. 2C, the scale of RF4 parameter is very small.

When the flight was finished, the pilots were asked to compare whether they were more stressed with the navigation or without. The result is that seven pilots reported that the navigation made them stressed and therefore they decided not to follow it. They also complained that it was not clear where the navigation led them and could not localize the landing place themselves. Some pilots decided to drop the navigation once the airfield was in reach and could see it. They did not follow the path for landing and claimed, that it would be enough if they just knew the direction in which the airport is, the airport’s heading, the distance, and the aircraft potential altitude on the moment of arrival. They also noted that they would not trust the system if it would not consider wind.

### Measurement

**Figure 3:**
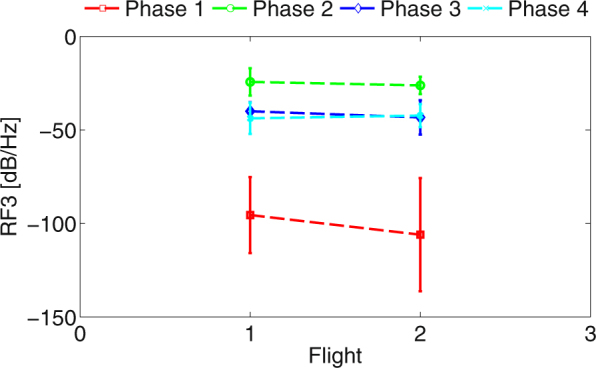
Flight type (1–navigation included, 2–navigation excluded) against flight phase (1–before engine failure, 2–engine failure, 3–glide, 4–approach and landing).

Physiological data measured during experiment were analyzed with ANOVA except of a Fig. 3. Figure 3 shows on raw data how parameter changed with respect to flight type. There can be seen no significant difference between the two flights. Phases 2 and 3 were of greatest interest, because in case that navigation would have some influence on physiology, it would be most likely seen here as a drop or increase of mean value between the flights.

**Figure 4:**
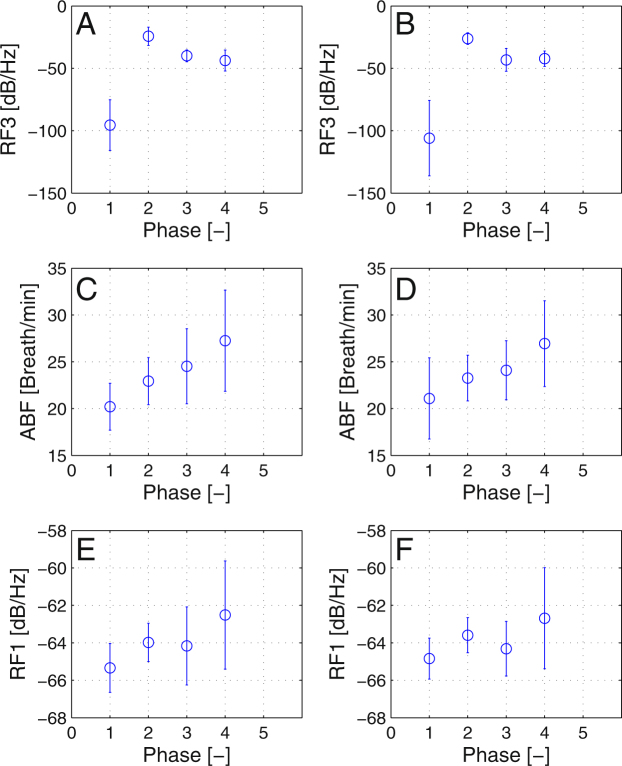
Means and standard deviations of statistically significant parameters against flight phase (1–before engine failure, 2–engine failure, 3–glide, 4–approach and landing). The left column with figure (**A**,**C**), and (**E**) is for flights with navigation enabled, the figures (**B**,**D**), and (**F**) are for flights without navigation support.

Figure 4A,B are not significantly different in terms of flight with navigation and without it. On the other hand, there is a significant difference between flight phases with $p=9.6\times {10}^{-5}$. After providing multiple comparison test, the phase 1 (before engine failure) is significantly different to all other phases in the level of 0.05. The mean value for first phase is $R{F}_{1}=-\,100.74\,dB/Hz$ and for second phase $R{F}_{1}=-\,25.24\,dB/Hz$. Second, third and fourth phase means are not significantly different.

**Figure 5:**
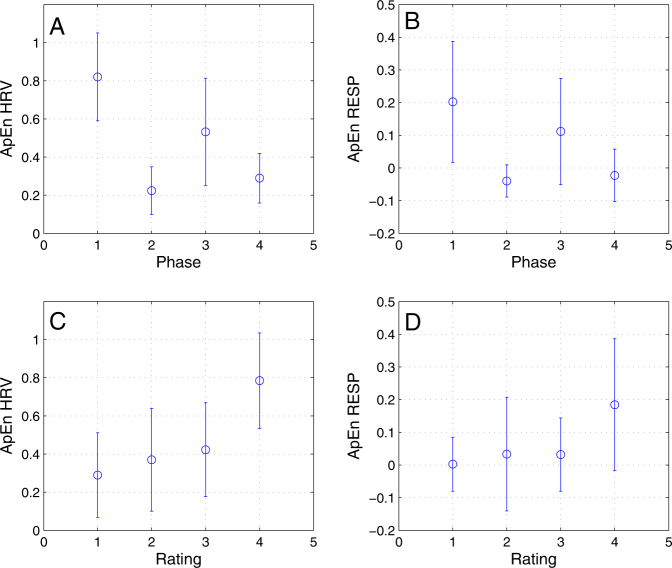
Means and standard deviations of approximate entropies of heart rate variability and breath frequency plotted against flight phase (1–before engine failure, 2–engine failure, 3–glide, 4–approach and landing) and subjective rating (1–most perceived stress, 4–least perceived stress).

From employed entropy measures, which were applied to HRV and respiration frequency are showed in Fig. 5 results of approximate entropy parameter. This was from all calculated entropy measures the only statistically significant. Based on multiple ANOVA the rating and phase has significant effect on the mean. The flight type again does not appear to have effect on the group mean. From multiple comparison tests it appears the variability related to phase suggests that phase 2 and 4 are both significantly different from phases 1 and 3 on the level of 0.05. Phase 1 and 3 are also significantly different on the same level of significance. HRV related to ratings shows, that the 1, 2, and 3 ratings have significantly different means from rating 4. This can be seen on Fig. 5A,C.

Approximate entropy of respiration analyzed with multiple ANOVA shows that phase groups do not have the same mean with $p=4.36\times {10}^{-7}$, and rating groups also do not have the same mean with *p* = 0.0022. From additional multiple comparison tests is seen that in appraisal the rating 4 has mean statistically different from the rest of ratings and the ratings 1 to 3 do not have significantly different mean. Phase groups 2 and 4 have significantly different mean from phase 1 and 3.

Average breathing frequency exhibits similar significant trend between phases with $p=2.2\cdot {10}^{-5}$ and no significant change in terms of flight as seen on Fig. 4C,D. The means of first (before engine failure) and last phase (approach and landing) are significantly different.

Parameter RF1 is significantly different only in the phase of approach and landing with *p* = 0.001.

Result from ANOVA are summarized in Table 1 listing significant parameters only. From all used parameters were observed only four to be significant.

**Table 1:** ANOVA analysis on the level of significance 0.05; n.s.–not significant.

Parameter	Dimension		
	Rating	Phase	Flight
RF1	n.s.	p < 0.05	n.s.
RF3	p < 0.05	p < 0.05	n.s.
RF4	p < 0.05	n.s.	n.s.
ABF	p < 0.05	p < 0.05	n.s.

## Discussion

From measured EDA, ECG, and respiration is extracted 19 parameters. EDA is excluded from analysis and together with additional HRV and respiration processing will be subject of another paper. Breathing parameters ABF, RF1, RF2, RF3, and RF4 are statistically significant. From six ECG parameters none is statistically significant in the context of current evaluation.

Navigation interface does not appear to have an effect on any measured physiological parameters. It seems pilots are experiencing very similar physiological states without regard to presence of a navigation assistant. It can have multiple reasons and it will be part of further analysis to assess the opinions of pilots about the navigation instrument.

On the other hand, subjective stress assessment has significant results for ABF, RF3, and RF4. ABF and RF3 follow similar pattern. The first three most stressful ratings yield similar means, but the rating least stressed is significantly different. The same case happens for RF3. It suggests that pilots might experience some sort of discomfort or stress in case of ratings 1 to 3, but for rating 4 they did not feel stressed. Rating 2 in Fig. 2C has significantly lower power than other three ratings, suggesting, that despite the subjects claiming to feel stressed, they actually were not. Figure 2C in the context of Figs 3 and 4E,F has very small means and therefore is not considered relevant to the evaluation despite its statistically significant results.

In terms of phase of flight, for ABF phase approach and landing (phase 4) is significantly different from the rest of phases. Same applies for parameter RF1. Parameter RF3 has phase 1 significantly different from the rest of phases. All above mentioned parameters share, that first and fourth phase are significantly different. Phase four has an increase of breathing frequency and increase in power for bands RF1 and RF3, which suggests increased demands on pilot or stressful experience. Since approach and landing is the most critical part of flights, it is expected to measure increased values.

To summarize the results, it seems that with the moment of engine failure pilots experienced physiological changes, which might be related to increased demands on pilot to control gliding plane, and to experiencing stress. Unfortunately the flight with navigation and without it did not provide any significantly different results. It seems that navigation instrument in this form does not have an effect on stimulation of pilots stress or load. Evaluation of ratings suggests most and least stressful phases of flight are significantly reflected in measured parameters.
